# Multi-parametric PET/MRI for enhanced tumor characterization of patients with cervical cancer

**DOI:** 10.1186/s41824-022-00129-2

**Published:** 2022-04-05

**Authors:** Sahar Ahangari, Flemming Littrup Andersen, Naja Liv Hansen, Trine Jakobi Nøttrup, Anne Kiil Berthelsen, Jesper Folsted Kallehauge, Ivan Richter Vogelius, Andreas Kjaer, Adam Espe Hansen, Barbara Malene Fischer

**Affiliations:** 1 Department of Clinical Physiology, Nuclear Medicine and PET, Rigshospitalet, University of Copenhagen, Copenhagen, Denmark; 2 Department of Clinical Medicine, University of Copenhagen, Copenhagen, Denmark; 3 Department of Oncology, Section of Radiotherapy, Rigshospitalet, University of Copenhagen, Copenhagen, Denmark; 4 Danish Centre for Particle Therapy, Aarhus University Hospital, Aarhus, Denmark; 5 Cluster for Molecular Imaging, Department of Biomedical Sciences, University of Copenhagen, Copenhagen, Denmark; 6 Department of Diagnostic Radiology, Rigshospitalet, University of Copenhagen, Copenhagen, Denmark

**Keywords:** Multi-parametric imaging, PET/MRI, Tumor heterogeneity, Radiotherapy, Cervical cancer, DWI, PET, RGD

## Abstract

**Aim:**

The concept of personalized medicine has brought increased awareness to the importance of inter- and intra-tumor heterogeneity for cancer treatment. The aim of this study was to explore simultaneous multi-parametric PET/MRI prior to chemoradiotherapy for cervical cancer for characterization of tumors and tumor heterogeneity.

**Methods:**

Ten patients with histologically proven primary cervical cancer were examined with multi-parametric ^68^Ga-NODAGA-E[c(RGDyK)]_2_-PET/MRI for radiation treatment planning after diagnostic ^18^F-FDG-PET/CT. Standardized uptake values (SUV) of RGD and FDG, diffusion weighted MRI and the derived apparent diffusion coefficient (ADC), and pharmacokinetic maps obtained from dynamic contrast-enhanced MRI with the Tofts model (iAUC_60_, *K*^trans^, *v*_e_, and *k*_ep_) were included in the analysis. The spatial relation between functional imaging parameters in tumors was examined by a correlation analysis and joint histograms at the voxel level. The ability of multi-parametric imaging to identify tumor tissue classes was explored using an unsupervised 3D Gaussian mixture model-based cluster analysis.

**Results:**

Functional MRI and PET of cervical cancers appeared heterogeneous both between patients and spatially within the tumors, and the relations between parameters varied strongly within the patient cohort. The strongest spatial correlation was observed between FDG uptake and ADC (median *r* =  − 0.7). There was moderate voxel-wise correlation between RGD and FDG uptake, and weak correlations between all other modalities. Distinct relations between the ADC and RGD uptake as well as the ADC and FDG uptake were apparent in joint histograms. A cluster analysis using the combination of ADC, FDG and RGD uptake suggested tissue classes which could potentially relate to tumor sub-volumes.

**Conclusion:**

A multi-parametric PET/MRI examination of patients with cervical cancer integrated with treatment planning and including estimation of angiogenesis and glucose metabolism as well as MRI diffusion and perfusion parameters is feasible. A combined analysis of functional imaging parameters indicates a potential of multi-parametric PET/MRI to contribute to a better characterization of tumor heterogeneity than the modalities alone. However, the study is based on small patient numbers and further studies are needed prior to the future design of individually adapted treatment approaches based on multi-parametric functional imaging.

## Background

Advanced medical imaging techniques can extract quantitative metabolic and physiological measures which reflect pathophysiology (O’Connor et al. 2017). Such multi-parametric data has the potential to enable a more individualized approach to cancer treatment (Beaton et al. 2019). Radiation therapy (RT) has evolved to be one of the essential therapies for cancer treatment. Recent advancement in radiation techniques enables a more personalized treatment planning and facilitates conformal dose distributions with improved target volume coverage (Dröge et al. 2021). Cervical cancer is a common malignancy among young and middle-aged women, with radiotherapy being a cornerstone in treatment. Despite advances in radiotherapy planning and dose delivery, there is still room for improving local control. One fourth of patients with cervical cancer developed a cancer recurrence and about 24% of the recurrences occur within the treated volume (Kobayashi et al. 2016; Lin et al. 2021; Pötter et al. 2021), indicating that there is still room to identify radio-resistance areas within a tumor that require radiation dose escalation. Therefore, the availability of robust and validated biomarkers is essential for accurate target volume definition during the radiotherapy planning process and holds a great demand for more advanced medical imaging techniques (Gatenby et al. 2013).

High-resolution structural MRI is conventionally used in combination with CT for evaluating the local extent of the tumor and the involvement of local lymph nodes in cervical cancer (Fields and Weiss 2016; Lim et al. 2011). However, functional tissue properties acquired from PET and MRI may correlate better with the true extent of tumor (Schwartz et al. 2018). Diffusion weighted (DW) MRI has been found to reflect cell density and correlates with tumor microstructure and heterogeneity (Le 2013; Liu et al. 2019; Szczepankiewicz et al. 2016). It is considered a promising tools for radiotherapy to define areas of tumor aggressiveness based on tumor cellularity (Tsien et al. 2014) and a useful indicator to predict the risk of disease recurrences and early therapeutic response for cervical cancer (Watanabe et al. 2021; Gladwish et al. 2016; Harry et al. 2021). Dynamic contrast enhanced (DCE) MRI is a non-invasive marker of tumor microcirculation and has shown potential for treatment outcome prediction (Mannelli et al. 2010; Dappa et al. 2017; Lee et al. 2015) and visualizing treatment-resistant regions in cervical cancer (Torheim et al. 2016). Further, quantitative measurement of DCE-MRI has been shown to correlate with vascular permeability and angiogenesis which is associated with tumor aggressiveness and clinical outcome (Hylton 2006). Recently, a new PET tracer, ^68^ Ga-NODAGA-E[c(RGDyK)]2 ([^68^ Ga]Ga-RGD), has been developed for imaging of α_v_β_3_, which is a type of integrin expressed in newly formed endothelial cells of the vasculature. This tracer is promising for the assessment of angiogenic activity in relation to cancer (Danhier et al. 2012). One study found a spatial relation between perfusion and α_v_β_3_ expression (Metz et al. 2010).

Multi-parametric analysis of PET and MRI data in radiotherapy has gained attention lately (Even et al. 2016; Daniel et al. 2017) with high potential in defining tumor sub-volumes for treatment individualization. With the advent of hybrid PET/MRI, it is feasible to perform one stop-shop PET/MRI acquisition of the pelvic region in RT treatment position (Ahangari et al. 2021). Recent studies have associated different functional and molecular information of PET and MRI to investigate the potential for a synergistic combination of image information that could lead us toward personalized patients care (Leibfarth et al. 2016; Esfahani et al. 2021; Shih et al. 2021). The combined analysis of multi-parametric images have been conducted to identify tumor sub-volumes in other anatomical regions (Even et al. 2017; Rasmussen et al. 2017), but not in cervical cancer. It should also be mentioned that RGD-PET has never been evaluated in combination with other parameter maps in pelvis region. However, different areas of tumor heterogeneity based on the tracer uptake was evaluated in lung cancer (Metz et al. 2010). In addition, multi-parametric images for target definition can decrease interobserver variability (Han et al. 2016; Lai et al. 2017). More advanced approaches have established a predictive model on multiple parameters to measure voxel-wise probability of tumor recurrence (Lundemann et al. 2019; Gao et al. 2020).

The aim of this study is to explore the potential of multi-parametric hybrid PET/MRI with RGD-PET to non-invasively characterize tumor heterogeneity prior to chemoradiotherapy of patients with cervical cancer. We wish to examine the relation between several functional and molecular imaging parameters and explore if imaging of tumor angiogenesis, perfusion characteristics and tissue microstructure can be used to identify tumor sub-volumes and thus potentially contribute to a better understanding of the pathophysiology of cervical cancer and improved tumor delineation.

## Materials and methods

### Patient cohort

In this prospective study, we included 10 patients with histologically proven advanced stage primary cervical cancer. Details of the patient population are summarized in Table 1. All patients were planned for treatment with radio-chemotherapy according to the current GEC-ESTRO guidelines (Pötter et al. 2015). This treatment consists of standard external beam radiotherapy (EBRT) (45 Gy in 1.8 Gy daily fractions to the pelvis ± paraaortic lymph nodes), weekly cisplatin chemotherapy (40 mg/m^2^), followed by an MR-guided brachytherapy.

**Table 1:** Summary of patient characteristics, tumor volumes, and quantitative imaging parameter for all available datasets. Median value is reported for ADC map and pharmacokinetic parameters

Patient	Age (years)	Weight (kg)	Histological type	HPV	FIGO stage	Tumor volume (cm^3^)	FDG-PET SUV_mean_	RGD-PET SUV_mean_	ADC 10^−6^ (mm^2^/s)	*K*^trans^ (1/min)	*v*_e_	*k*_ep_ (1/min)
1	68	84	Squamous cell carcinoma	+	IIB	10.5	7.7	10.5	1097	128	144	80
2	29	54	Squamous cell carcinoma	+	IIIC	32.2	17.0	2.9	1199	78	96	79
3	65	76	Squamous cell carcinoma	Unknown	IIIC	30.5	9.8	4.7	941	104	151	65
4	68	78	Squamous cell carcinoma	+	IIB	20.3	9.5	6.4	1156	203	238	81
5	45	60	Adenocarcinoma	+	IB3	98.9	8.3	2.9	1176	139	141	94
6	60	70	Adenocarcinoma	+	IIIC1	125.8	15.7	5.1	1332	56	126	39
7	55	69	Squamous cell carcinoma	+	IIIC1	60.5	8.1	5.7	1368	156	207	73
8	41	100	Squamous cell carcinoma	+	IIIC1	120.3	11.5	NAN	935	89	130	66
9	46	65	Adenocarcinoma	+	IIIC1	55.9	7.8	6.3	1122	134	230	57
10	74	54	Squamous cell carcinoma	+	IIIB	32.1	5.9	7.1	991	90	167	58

All patients gave written informed consent, and the study was approved by the Regional Ethics Committee (ref. no. H-18042903).

### Multi-parametric imaging

#### FDG-PET/CT

Patients received a PET/CT scan as part of the routine staging procedure. The PET/CT scans were carried out after intravenous injection of ≈ 3 MBq/kg 18F-FDG on a Biograph Vision 600 PET/CT scanner (Siemens Healthcare, Erlangen, Germany). CT was performed first, with a tube voltage of 120 kV and reference mAs of 240 using CareDose 4D. The whole-body PET data was acquired with an acquisition time of 3 min per bed position covering the proximal thigh to the base of the skull. FDG-PET image reconstruction was performed using 3D-OP-OSEM algorithm including Time of Flight, 4 iterations, 5 subsets, and a Gaussian filter of 2 mm FWHM.

#### RGD-PET/MRI

PET/MRI examinations were carried out on a whole-body hybrid PET/MRI system (Biograph mMR, Siemens, Germany), 30 min after injection of 200 MBq ^68^ Ga-NODAGA-E[c(RGDyK)]_2_ (RGD). During the scanning procedure, patients were positioned feet-first supine in the RT treatment position. The patient setup ﻿has been evaluated and described in detail in our previous work (Ahangari et al. 2021).

PET data were acquired as a single-bed over the pelvis region for 20 min and images were reconstructed with 3D-OP-OSEM (3 iterations and 21 subsets, a Gaussian filter with 4 mm FWHM) on 344 × 344 image matrix with pixel size 2.1 × 2.1 mm^2^ and a slice thickness of 2 mm. The images were corrected for signal attenuation using the Dixon volumetric interpolated breath-hold sequence (Dixon-VIBE). Simultaneously with PET, high resolution T1 and T2 weighted turbo spin echo sequences (TSE) were acquired as part of the clinical routine for tumor delineation.

In addition, diffusion weighted single shot echo planar sequence was obtained over the cancer region with b values of 0 and 1000 s/mm^2^ (Liu et al. 2019). Apparent diffusion coefficient (ADC) maps were generated after the data acquisition from the vendor software (Syngo MR). For DCE-MRI imaging, a body-weight adapted dosage of gadolinium-DTPA contrast agent (0.1 ml/kg) was injected. Four axial pre-contrast T1-weighted VIBE sequence with various flip angles were acquired to measure T1 mapping for extracting DCE-MRI parameters. It was followed by the dynamic acquisition of fat saturated T1-weighted VIBE sequence in 84 frames and 2.9 s temporal resolution. Details of the entire imaging protocol are given in Table 2.

**Table 2:** Detailed PET/MRI scan protocol

PET	One BP PET (20 min)				DW-MRI (*b* = 0, 1000) RESOLVE	TrueFISP	DCE-MRI	
MRI	T1 Dixon VIBE	T2-W TSE	T2-W TSE	T1-W TSE			T1 VIBE	T1 VIBE + Gd
Orientation	Axial	Sagittal	Axial	Axial	Axial	Axial	Axial	Axial
TR (ms)	3.85	3840	3290	682	4040	382	5.18	5.18
TE (ms)	1.23,2.46	94	115	11	55,80	1.68	1.09	1.09
Matrix size	312 × 384 × 88	512 × 512 × 42	512 × 512 × 83	512 × 512 × 53	120 × 160 × 24	412 × 512 × 80	128 × 128 × 16	128 × 128 × 16
Pixel size (mm^2^)	1.3 × 1.3	0.48 × 0.48	0.48 × 0.48	0.48 × 0.48	2.5 × 2.5	0.74 × 0.74	2.5 × 2.5	2.5 × 2.5
Slice thickness (mm)	3	3	3	3	3	5	5	5
Flip angle (°)	10	160	160	160	180	45	2, 5, 15, 25	15

*VIBE* volumetric interpolated breath-hold, *TSE* turbo spin-echo, *BP* Bed position

### Pharmacokinetic modeling

Tissue perfusion based on DCE-MRI datasets were analyzed using Tissue 4D software (Siemens Healthineers, Erlangen, Germany). First, 3D motion correction was performed to verify the alignment of the dynamic dataset to a defined reference volume. For one patient, the last 17 time frames were deleted due to movement and insufficient quality for validation. T1 mapping was automatically calculated using the VIBE sequences with varying flip-angles. T1 mapping were incorporated to convert the signal to gadolinium concentration–time plots. The pharmacokinetic Tofts model was fitted on a voxel basis incorporating an arterial input function (AIF) to generate parameter maps of volume transfer coefficient (*K*^trans^), the rate constant between extracellular extravascular space and plasma (*k*_ep_), fractional volume of the extravascular-extracellular space (*v*_e_), and the initial Area-Under-Curve (iAUC_60_) for the first 60 s (Tofts et al. 1999). A region of Interest (ROI) was manually placed inside the iliac artery within FOV and the optimal AIF was selected using chi2 parameter from three population-averaged options (slow, intermediate, fast).

### Image registration

#### Cross-modality registration

The FDG-PET/CT images were moved into RGD-PET/MRI space by rigidly registering the CT to MRI. The registrations were performed in two steps using 6-parameter rigid alignment procedure (Minc toolkit, McConnell Brain Imaging Centre, Montreal, Canada). The initial alignment was performed to align pelvic bones using selected anatomical landmarks. Second, the local registration was performed using another set of landmarks with spatial attention to the cervix. Landmarks were modified until the root‐mean‐square distance between each landmark pair was minimized.

#### Intra-modality registration

To achieve optimal registration and account for bladder filling variation and tumor displacement during the scan, multi-parametric MR images were locally and rigidly registered to the anatomical T2-weighted MR images with great care given to the initial gross tumor volume. Diffusion weighted sequence with b value of 0 and the initial time frame of DCE-MRI were chosen a for registration. The relevant transformation was applied to each parameter map.

### Delineation

A delineation of the primary tumor was needed to perform voxel-wise analysis of parameter maps. To ensure the accuracy, the primary gross tumor volume (GTV) of each patient was manually delineated by an experienced radiologist (> 20 years clinical experience in cervix cancer) using standard routine software, Varian Eclipse. The local tumor extent was determined on axial T1-weighted and T2-weighted as well as sagittal T2-weighted images.

### Analysis

Image Analyses were performed using Python 3.6. Tumor volume was measured as the volume of GTV for each patient. For the respective dataset, the GTV was transferred to the parameter maps, and median values for ADC, b1000, and pharmacokinetic parameters within the tumor were determined. The corrected PET images were converted to standardized uptake values to reduce the dynamic range of the voxel intensity. Mean and maximum values for SUV_FDG_ and SUV_RGD_ were calculated to quantify the tumor uptake, where 50% threshold of maximum SUV was applied to the tumor voxels.

For correlation analysis, all functional and molecular parameter maps were resampled to the RGD-PET image. Voxel-by-voxel correlation analysis was performed for all available pairwise combinations of functional and parametric maps within the tumor. Correlations between parameters were quantified by creating an averaged color-coded Spearman’s rank correlation coefficient matrix on a voxel level.

Moreover, the GTV mask was applied to the parameter maps of each patient, and the voxels within the mask were pooled over all subjects. Joint histogram of parameter maps were computed across pooled data using 120 × 120 bins. All voxels within the tumors were assigned to the corresponding histogram bins and the intensity of the bins represents the relative frequency of the voxels within a specific range of parameters. The information being captured using joint histograms were used to guide selection of parameters for cluster analysis. The ability of multi-parametric data to characterize different tumor microenvironments was tested on selected parameters using an unsupervised 3D Gaussian mixture model-based clustering (GMM) on pooled data. The combined dataset was normalized, and all voxels were divided into three clusters by GMM. The percentage of the tumor sub-volume belonging to each cluster was obtained for individual patients.

## Results

All patients completed the imaging protocol successfully and the images were suitable for further evaluation. The total duration of the PET/MRI examination was 47 min in average. For one patient, it was not possible to administer RGD due to a tracer production issue. This patient was excluded from the cluster analysis.

**Fig. 1:**
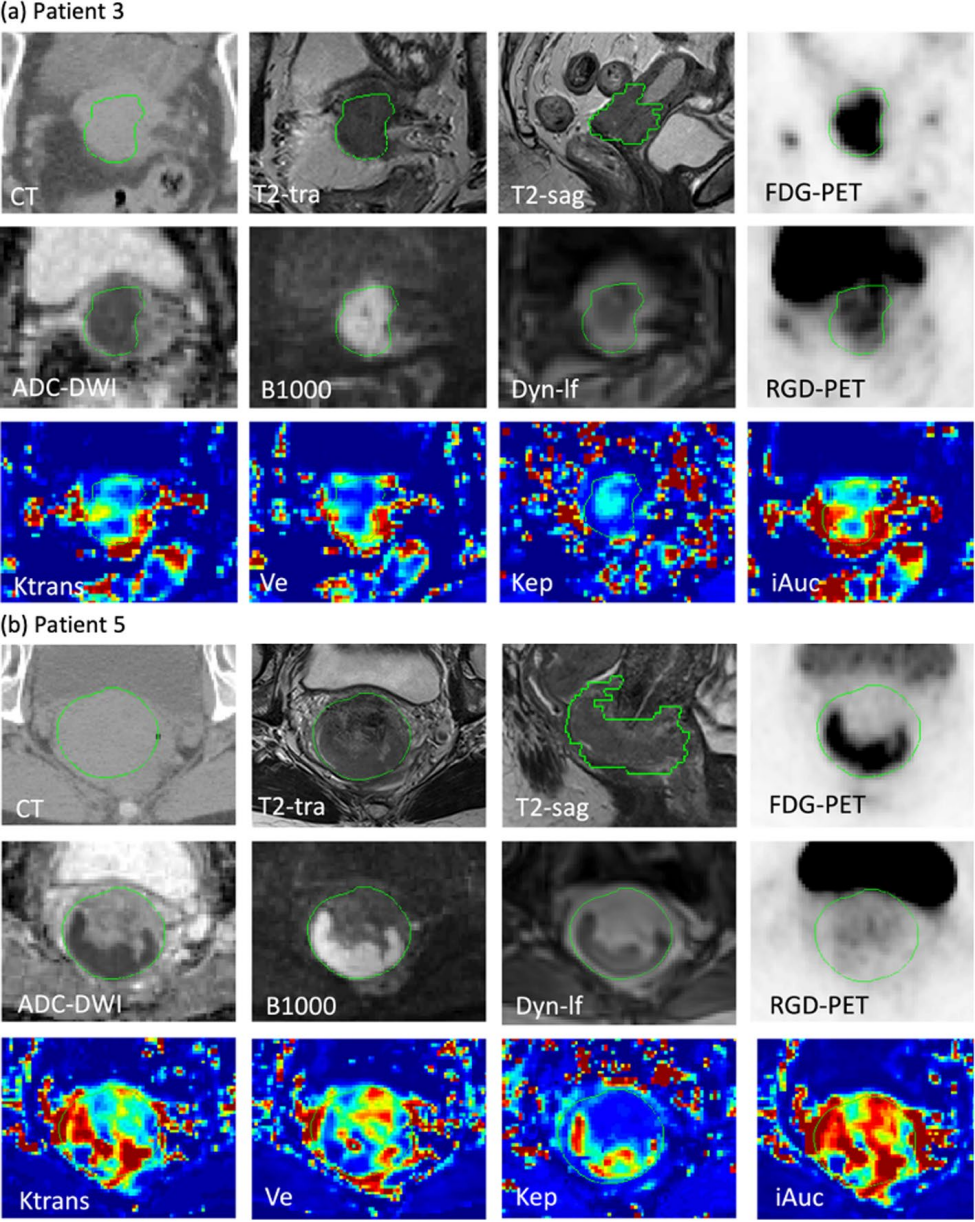
Representative examples of co-registered parametric maps for patient 3 (**a**) and patient 5 (**b**). From upper left to lower right: planning CT, axial and sagittal T2-weighted MRI, FDG-PET, diffusion parameters (ADC, B1000), last frame of DCE, RGD-PET, and perfusion parameters (*K*^trans^, *v*_e_, *k*_ep_, and iAUC_60_). Green contour represents GTV

Seven of ten patients were diagnosed with squamous cell carcinomas and three with adenocarcinoma. Anatomical MRI images of all patients were reviewed and approved by an oncologist and a radiologist before incorporating them into the clinical workflow. The co-registered image datasets including all functional images and parameter maps and the delineated GTV are visualized for two representative patients with different tumor types in Fig. 1. There has been observed a different level of intra-tumor heterogeneity of all parameters for patients. Qualitatively, all parameter maps show intra-tumor heterogeneity, but the degree of heterogeneity varies between patients.

Table 1 summarizes the characteristics of all patients as well as a complete overview of the respective imaging parameters, expressed as mean values of SUV uptake and median values of diffusion and perfusion measurements within the tumor. The average tumor volume was 58.7 ± 39.9 cm^3^. All tumors were highly FDG-avid with average SUV_mean_ and SUV_max_ of 10.1 ± 3.5 and 15.2 ± 5.4 respectively. The average SUV_mean_ and SUV_max_ for RGD-PET were 5.7 ± 2.2 and 8.2 ± 4.5 respectively. However, the RGD-uptake was more heterogeneous between patients with a very low RGD uptake (SUV_mean_ < 3) in two patients and a relatively high RGD uptake in one patient (SUV_mean_ = 10.5). All tumors demonstrated restricted diffusivity, with average ADC values of mean 1131 ± 141 × 10^−6^ mm^2^/s.

**Fig. 2:**
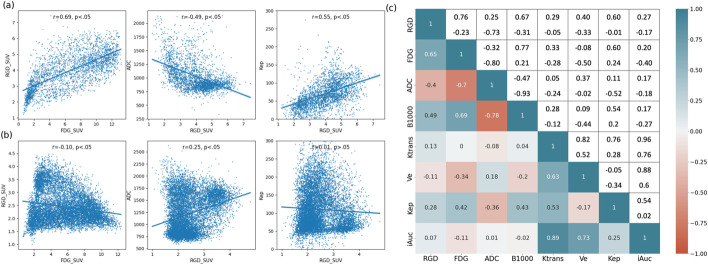
Representative scatter plots for patient 3 (**a**) and patient 5 (**b**), showing voxel-wise distribution of selected pairs of parameters. On the right side (**c**), the correlation matrix of median Spearman correlation coefficient between all investigated parameters (lower-left triangle) and its range across all patients (upper-right triangle)

Results of the voxel-based pairwise correlation analysis are shown in Fig. 2. In Fig. 2a, b, the voxel-wise relation of FDG and RGD uptake, RGD uptake and ADC, and RGD uptake and *k*_ep_, is shown for the same two patients as in Fig. 1. The scatter plots for patient 3 show moderate correlations, which are positive between FDG and RGD uptake and between RGD uptake and *k*_ep_, and negative between RGD uptake and ADC. The voxel wise distribution of ADC and RGD-PET appears L-shaped rather than a direct negative correlation. However, for patient 5 the correlations for the same pairs of parameters are fairly weak and of opposite sign. Figure 2c displays a correlation matrix on the lower left triangle representing the median Spearman correlation coefficients obtained from all tumor voxels in all available patient datasets and for all pairwise combinations of parameter maps. The upper right triangle indicates the range of Spearman correlation coefficients obtained in each patient. The voxel-based pairwise correlation coefficients vary strongly between patients. Except the internal correlation between perfusion parameters, the strongest correlation is between FDG uptake and ADC (median *r* = -0.7, range from − 0.32 to − 0.80). RGD uptake appears to have moderate correlation with FDG uptake (median *r* = 0.65, range from − 0.23 to 0.76) and ADC (median *r* = -0.4, range from − 0.73 to 0.25). There are relatively weak correlations between perfusion parameters and all other parameters.

**Fig. 3:**
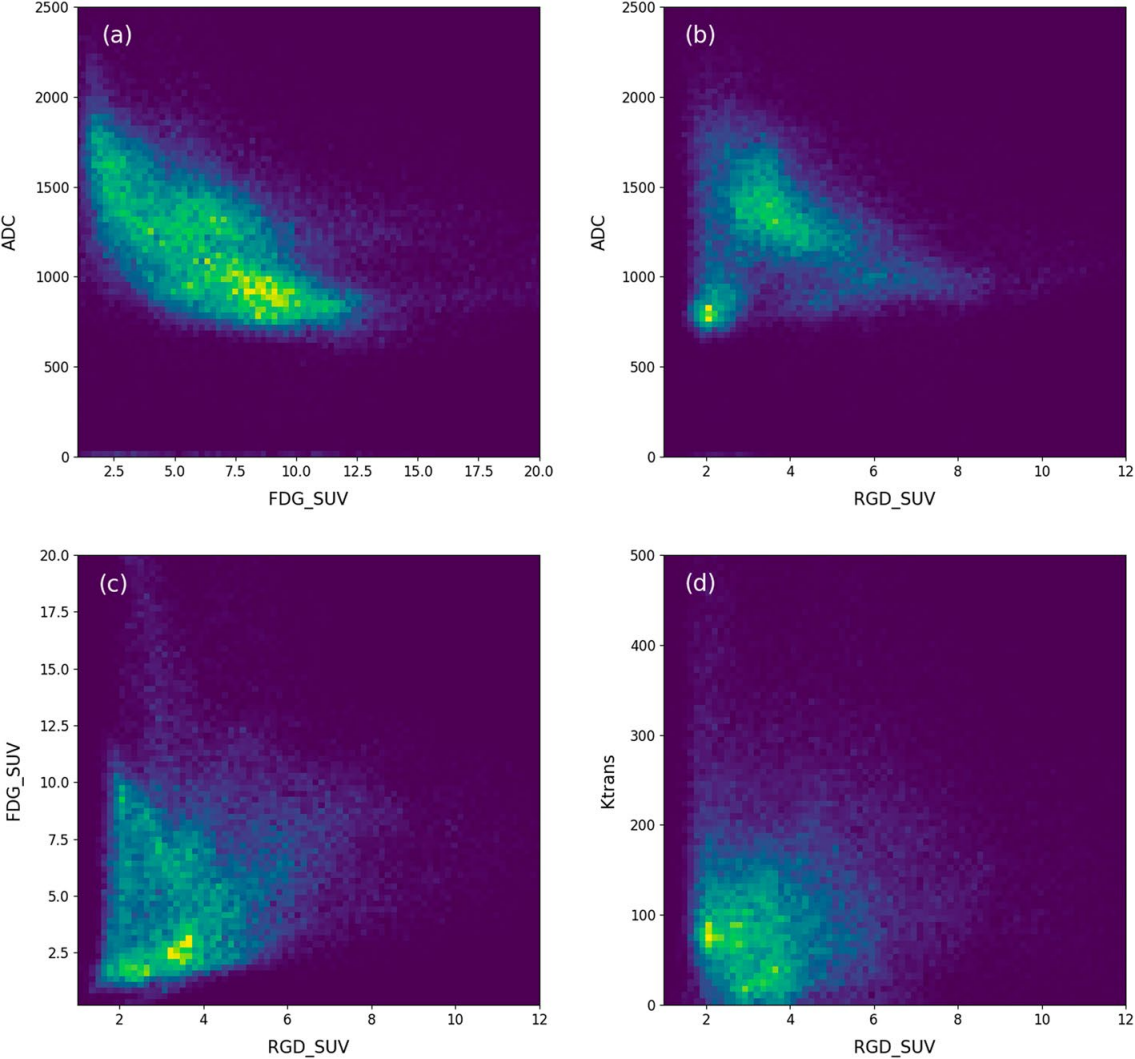
Joint histograms across all voxels, between FDG-PET and ADC (**a**), RGD-PET and ADC (**b**), FDG-PET and RGD-PET (**c**), and RGD-PET and *K*^trans^ (**d**)

**Fig. 4:**
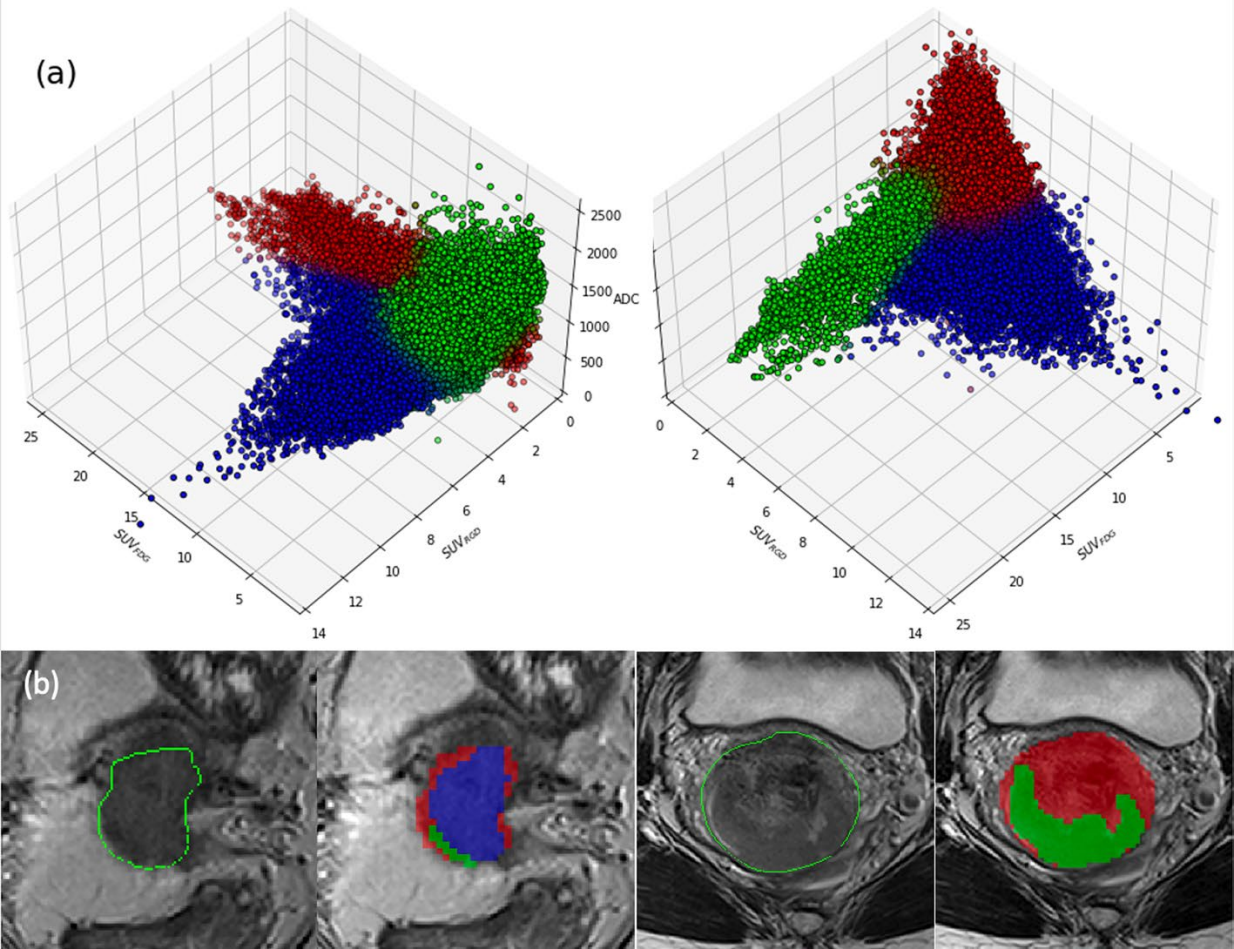
3D scatter plot of the multi-parametric data representing the clusters fitting with a Gaussian mixture model (**a**). The clusters are mapped back to the T2-weighted MRI images for two representative patients (**b**)

Figure 3 shows the joint histogram of FDG-PET and ADC, RGD-PET and ADC, FDG-PET and RGD-PET, and RGD-PET and *K*^trans^. In summary, Fig. 3a demonstrates a negative intra-tumoral relationship between FDG uptake and ADC values. The same negative relation is observed between RGD uptake and ADC in Fig. 3b, except for a separate, well-defined cluster of voxels with low ADC and low RGD uptake. The distribution of the RGD-PET values appears to have no clear relation with FDG uptake and *K*^trans^. As the joint histograms of FDG uptake and ADC, and RGD uptake and ADC show the most distinct structures, those parameters were chosen for a cluster analysis. A 3D representation of all tumor voxels is illustrated in Fig. 4, showing the relationship between ADC, FDG and RGD uptake. The points are colored according to the Gaussian probability of belonging to the cluster. Figure 4b represents the segmentation of the tumor lesion into sub-volumes as defined by the cluster analysis for two representative patients.

## Discussion

In the present study, a multi-parametric PET/MRI scan protocol was suggested to non-invasively characterize tumor heterogeneity in patients with cervical cancer. The heterogeneity in tumor was explored using voxel wise analysis of MRI measurement of diffusion and perfusion parameters as well as PET measurement of angiogenesis and glucose metabolism. The relationship between the imaging modalities in this comprehensive set of functional data suggests a proof of concept with potential insight into tumor pathophysiology. Further studies, in particular in combination with survival analysis and biopsy verification, are needed to shed light on the clinical and pathophysiological relevance of these relationships. We may speculate, that multi-parametric imaging and analysis can in the future be used for individual therapy adaptation with inhomogeneous radiation dose prescription.

Functional MRI and PET parameter maps appeared heterogeneous within the tumors, and the degree of heterogeneity, as well as the relations between parameters, varied between patients. Tumor heterogeneity is a common feature of the tumor microenvironment and heterogeneity as captured by medical imaging could be an important factor in identifying high-risk volumes as well as prognosis (Mayr et al. 2012; Davnall et al. 2012).

Tumors were clearly delineated by DW-MRI. The heterogeneity of ADC maps could be attributed to increased diffusion restriction by tumor cells, while cystic and necrotic tissues show higher ADC values. Our findings support the potential of DW-MRI for target volume delineation, as has been described in depth in several studies (Dappa et al. 2017; Han et al. 2016). The use of a b-value of zero may result in overestimation of the ADC due to contributing perfusion effects (Tsien et al. 2014). However, it is commonly used in the setting of cervical cancer imaging (Liu et al. 2019; Watanabe et al. 2021; Daniel et al. 2017).

A moderate to high level of FDG uptake was observed across patients. On the other hand, the patients show a very variable level of RGD uptake including two patients had very low RGD uptake. FDG-PET is widely used as a surrogate parameter of cell viability and glucose metabolism (Pötter et al. 2015; Caresia-Aróztegui et al. 2019), while RGD uptake is used to assess integrin α_v_β_3_ expression and angiogenic tumor cells (Durante et al. 2020). The heterogeneity of both FDG and RGD uptake within the same cancer type could be due to differences in properties such as growth rate, vascularity and necrosis in the tumor cell population (Metz et al. 2010). In an explorative analysis, we found no relation of RGD and FDG uptake to histological tumor type and tumor grade (Table 1).

Among other frequently used pharmacokinetic models, the standard Tofts model has been reported to be preferable in analysis of clinical DCE-MRI data (Litjens et al. 2010). The parameters estimated using the Tofts model had a high degree of variation within the patient cohort. The volume transfer constant *K*^trans^ is determined by the blood perfusion and the permeability surface area product of the vessel wall in varying proportions and expected to correlate with RGD uptake as both parameters are supposed to represent angiogenesis and active endothelial cells (Dappa et al. 2017; Liu 2009). However, we did not observe any significant relation between perfusion metrics and other parameters. This finding is contrary to previous observation by Metz et al. (Metz et al. 2010), wherein they found a tendency toward higher values of tumor perfusion in areas with more intense RGD uptake. The results are also not supporting the claim that tumor perfusion could be simulated by FDG uptake (Metz et al. 2010), which may occur when the tumor blood flow is inadequate and requires a well-developed tumor vascular supply.

Except for one patient with a small tumor (volume of 10.5 cm^3^), the RGD uptake was generally lower than FDG which is in line with previous results for lung cancer (Durante et al. 2020). The voxel-by-voxel analysis of parameter maps revealed weak correlations between RGD and FDG uptake, which varied strongly within the patient cohort, indicating that they provide different and potentially complementary information about the tumor. Our results demonstrated an average correlation coefficient of − 0.7 between FDG uptake and ADC values, in line with earlier studies (Leibfarth et al. 2016; Olin et al. 2018; Olsen et al. 2013), while the correlation between ADC and RGD uptake was weaker.

On a voxel-wise level, there appear to be an L-shaped relation of FDG uptake and ADC values in the 2D joint histogram (Fig. 3a). This type of relation between ADC and FDG uptake has been widely observed for different regions and cancer types (Gong et al. 2019; Steiner et al. 2021). The same trend can be observed between RGD uptake and ADC (Fig. 3b). However, the 2D histogram analysis suggests a cluster of tumor voxels with low ADC and low RGD uptake. The majority of the voxels corresponding to this area belong to patient 2 and patient 5, both having tumors with very low RGD uptake. We went one step further to evaluate the combination of ADC, FDG, and RGD uptake and explore their ability to identify tumor sub-volumes. Distinct clusters in Fig. 4, resulting from GMM model were indicative of a viable tumor region (blue dots with high RGD uptake, intermediate FDG uptake, and low ADC value), and a region with mixed necrosis (red dots with low RGD, low FDG, high ADC). The third cluster however is more equivocal with low ADC values, variable FDG uptake and almost no RGD uptake (green dots). The third cluster corresponds to the previously referred to feature in the joint histogram of ADC and RGD uptake (Fig. 3b, lower left). We may speculatively interpret the third cluster as representing solid tumor tissue however with a small number of newly formed vessels. Tissue histology will be needed to shed more light on the possible significance of the cluster analyses. A cluster analysis has been done before in a preclinical study considering FDG and ADC to provide a more stable metric for multi-parametric tumor characterization (Divine et al. 2015) A similar study was conducted on patients with non-small cell lung cancer (NSCLC) (Metz et al. 2015). They created 4 clusters based on single value as a threshold to distinguish between high and low ADC value and FDG uptakes.

In studies employing multi-parametric imaging, alignment between different modalities will always be a potential limitation. It highlights the major advantages of combined PET/MRI scanner in the more straightforward voxel-by-voxel analysis of MR and PET parameters. However, it is still a critical issue for studies of the pelvic area, due to changing patient anatomy in particular originating from urinary bladder filling. Depending on the time interval between acquisition of each image as well as the proximity of the tumor to the bladder, it can cause misalignment over tumor volume. Our solution to this issue to manually perform local registration between images could reduce the effect.

The analysis of voxel-wise correlations required resampling of images to the same voxel size. The reconstructed PET had the highest resolution of the multi-parametric images and was chosen as common voxel size, in order not to lose image information. The resulting upsampling of DWI and DCE-MRI may result in inaccuracies of the correlation analysis. For the multi-parametric imaging analysis in the tumors, the radiotherapy target delineation was employed, as contoured by an experienced radiologist. To ensure reproducibility of a potential future studies and implementation, a semi-automated or deep-learning approach to tumor delineation could be explored.

It needs to be stated that the current results are based on a low number of patients. The focus of this study, however, was to explore feasibility and the potential of multi-parametric PET/MRI for cervical cancer. Thus, it might contribute to the future design of individually adapted treatment approaches based on multi-parametric functional imaging. Studies with a larger cohort are demanded to further evaluate our findings. The correlation analysis of parameter maps with histopathology should be explored in the future to evaluate the potential of multi-parametric imaging for delineation reproducibility and enhanced tumor characterization.

## Conclusions

Multi-parametric PET/MR in patients with cervical cancer is feasible and it provides a substantial amount of complimentary functional imaging data, which may be beneficial for cancer treatment adaptation. Our suggested hybrid PET/MRI protocol allows to non-invasively explore spatial pattern of tumor heterogeneity. The combined information of ADC, FDG- and RGD-PET images in tumors allows for segmentation into distinct tissue classes. However, future prospective studies on larger, homogeneous patient populations are needed to elucidate which combination of imaging parameters is best suited for tumor characterization.

## Data Availability

The datasets generated and/or analysed during the current study are not publicly available due the hospital policy.

## Abbreviations

ADC: Apparent diffusion coefficient
AIF: Arterial input function
DCE: Dynamic contrast enhanced
Dixon-VIBE: Dixon volumetric interpolated breath-hold sequence
DW: Diffusion weighted
EBRT: External beam radiotherapy
GTV: Gross tumor volume
GMM: Gaussian mixture model
*K*^trans^: Volume transfer coefficient
*k*_ep_: Rate constant between extracellular extravascular space and plasma
*v*_e_: Fractional volume of the extravascular-extracellular space
iAUC_60_: Initial area-under-curve
NSCLC: Non-small cell lung cancer
RGD: ^68^Ga-NODAGA-E[c(RGDyK)]2
ROI: Region of interest
RT: Radiation therapy
SUV: Standardized uptake values
TSE: Turbo spin echo sequences
